# Biochar can mitigate co-selection and control antibiotic resistant genes (ARGs) in compost and soil

**DOI:** 10.1016/j.heliyon.2022.e09543

**Published:** 2022-05-27

**Authors:** Chisom Ejileugha

**Affiliations:** Lancaster Environment Centre (LEC), Lancaster University, LA1 4YQ, United Kingdom

**Keywords:** Wastewater, Polyaromatic hydrocarbons, Biowastes composting, Antibiotic resistance, Environmental contamination, Heavy metals

## Abstract

Heavy metals (HMs) contamination raises the expression of antibiotic resistance (AR) in bacteria through co-selection. Biochar application in composting improves the effectiveness of composting and the quality of compost. This improvement includes the elimination and reduction of antibiotic resistant genes (ARGs). The use of biochar in contaminated soils reduces the bioaccessibility and bioavailability of the contaminants hence reducing the biological and environmental toxicity. This decrease in contaminant bioavailability reduces contaminants induced co-selection pressure. Conditions which favour reduction in HMs bioavailable fraction (BF) appear to favour reduction in ARGs in compost and soil. Biochar can prevent horizontal gene transfer (HGT) and can eliminate ARGs carried by mobile genetic elements (MGEs). This effect reduces maintenance and propagation of ARGs. *Firmicutes*, *Proteobacteria*, and *Actinobacteria* are the major bacteria phyla identified to be responsible for dissipation, maintenance, and propagation of ARGs. Biochar application rate at 2–10% is the best for the elimination of ARGs. This review provides insight into the usefulness of biochar in the prevention of co-selection and reduction of AR, including challenges of biochar application and future research prospects.

## 1 Introduction

Biochar is used in improving physicochemical and microbiological properties of soil (Guo et al., 2021; Kaur and Sharma, 2021) and compost (Akdeniz, 2019; Pandiyan et al., 2021; Yin et al., 2021). It is also used to remove contaminants (including antibiotics) from aqueous solutions and environmental media (Chen et al., 2021; Cheng et al., 2021). Environmental contamination influences antibiotic resistance (AR) and the emergence of AR in bacteria has remained a public health concern. The existence of antibiotic resistant bacteria (ARB) and the AR behaviour have been observed in both clinical and non-clinical samples (Baker-Austin et al., 2006; Goryluk-Salmonowicz and Popowska, 2019; Seiler and Berendonk, 2012). The AR is conferred on bacteria by genes existing in the chromosomes and the MGEs (Baker-Austin et al., 2006). The maintenance and propagation of ARGs in bacteria is affected by environmental contaminants through co-selection mechanisms and the impact of heavy metals (HMs) contamination on ARGs has received much attention (Mazhar et al., 2021; Ye et al., 2017; Zhao et al., 2019, 2020). The biowastes and biosolids used in composting can contain metals and antibiotics through the use of these products by humans and in animal management (Arya et al., 2021; Peng et al., 2021; Ye et al., 2017). Compost, biosolids, and biowastes are in use in soils as amendment and a long-term application of these products could lead to a high level of ARB in the environment.

Co-selection is the associated selection and expression of two or more resistance genes by bacteria even when exposed to only a selective trigger or stressor (Di Cesare et al., 2016). In addition to HMs (Baker-Austin et al., 2006; Gorovtsov et al., 2018; Maurya et al., 2020), other contaminants have been postulated to affect ARGs in the environment such as polyaromatic hydrocarbons (PAHs) (Gorovtsov et al., 2018), quaternary ammonium compounds (Wales and Davies, 2015), polychlorinated biphenyls (PCBs) (Gorovtsov et al., 2018), detergents (Wales and Davies, 2015; Ye et al., 2017), and anti-biofouling agents (biocides) (Wales and Davies, 2015; Ye et al., 2017). It is also postulated that ARB and ARGs may be higher in contaminated environmental media due to co-selection pressure (Cunningham et al., 2020).

**Figure 1:**
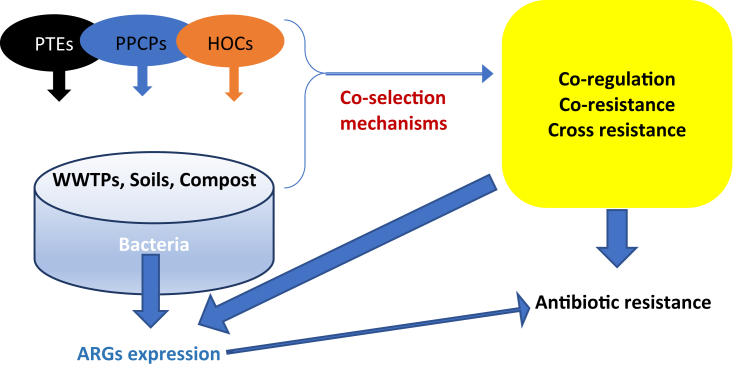
Environmental stressors, conditions, and co-selection mechanisms for antibiotic resistance.

Co-resistance, co-regulation, and cross resistance have been reported as strategies for co-selection in bacteria (Baker-Austin et al., 2006; Di Cesare et al., 2016; Maurya et al., 2020; Pal et al., 2017). In co-resistance, the genes coding for heavy metal resistance (HMR) and AR occurs together on the same MGEs like transposons, and plasmids. The genes are basically located (co-localized) on the same genetic element. In this situation, the expression or transfer of one gene, leads to the transfer of the other as both exist on the same carrier. In cross-resistance, resistance to a particular antimicrobial agent or antibiotic causes a resistance to all agents that share same mode of action. Cross-resistance refers to HMR and AR being expressed by the same bacterial gene through initiating a resistance mechanism which is against a mode of action shared by different stressors. This can be as a result of cell membrane permeability, efflux of agents, or modification of target sites. Co-regulation is linked to a situation where the existence of a given stressor, leads to an upregulation of certain gene which in turn prevents the impact of another stressor. For instance, the mechanism of resistance to a metal, is regulated by a gene, which is related to resistance to a given antibiotic. So, the expression of this gene in response to the metal stressor confers resistance to a given antibiotic. In other words, a gene which regulate the expression of a given HMR mechanism is also responsible for the regulation of a given AR mechanism, hence leading to a co-regulation of two mechanisms. Formation of biofilm can promote co-selection through co-existence and synergistic activities occurring in biofilm, which can lead to HGT of ARGs among bacterial species, hence conferring resistance and stability to the bacterial community (Baker-Austin et al., 2006). MGEs and HGT play a significant role in the proliferation and maintenance of ARGs (Di Cesare et al., 2016; Maurya et al., 2020). In addition, Class 1 integrons are also considered to play a major role in the propagation of ARGs in contaminated systems (Gaze et al., 2011; Maurya et al., 2020). Figure 1 shows the stressors, conditions and co-selection mechanisms that leads to ARGs expression and AR in the environment.

**Figure 2:**
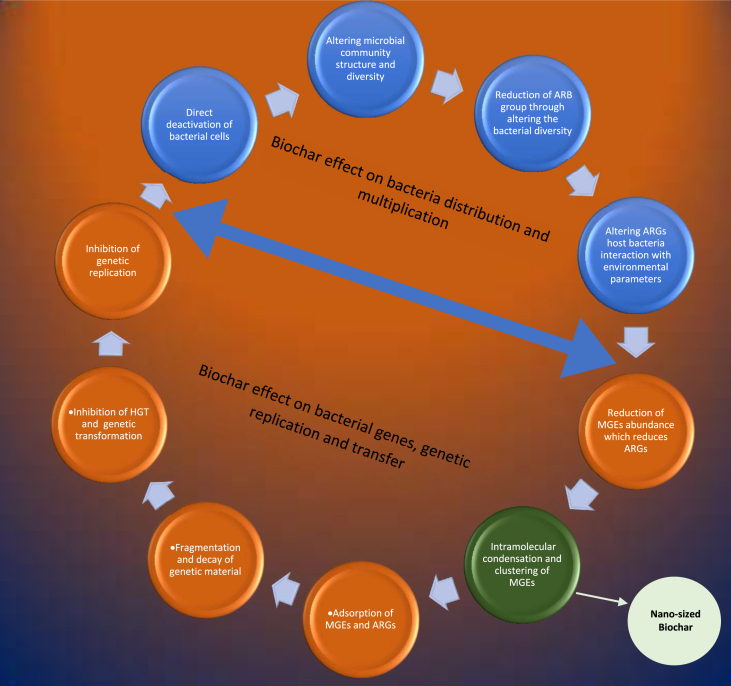
Mechanisms of biochar-ARGs elimination.

Bacteria can develop resistance to antibiotics through different resistance mechanisms. It have been proposed and reported in literature that the contamination of environmental media by metals can have a significant impact on the distribution and propagation of AR in bacteria (Cao et al., 2020a; Di Cesare et al., 2016; Wang et al., 2021b; Yang et al., 2020). Like antibiotics, heavy metals also possess antimicrobial properties (Maurya et al., 2020). Generally, antibiotic and metal resistance in bacteria share similar functional and structural mechanisms. The mechanisms of metal and antibiotic resistance in bacteria include (a) Reduction in cell membrane permeability to antibiotics and metals (b) Inactivation or alteration of antibiotic and metal (c) Efflux of metal and antibiotics (d) Cellular targets mutation or alteration (e) Sequestration of metal and antibiotic. Biochar eliminates ARGs through direct effects on bacteria and through affecting bacterial genes, genetic replication, and gene transfer. Figure 2 shows the various mechanisms involved in biochar elimination of ARGs in soil, composting, and wastewater treatment.

In recent times, there have been much emphasis on the impact of biochar on composting and compost quality (Akdeniz, 2019; Guo et al., 2020b; Sanchez-Monedero et al., 2018; Wu et al., 2017a). However, there is no review on the effectiveness of biochar in the control and elimination of ARGs in composting, soil, and wastewater treatment. This review gives insight into the use of biochar in the prevention of co-selection pressure and control of ARGs in compost, soil, and wastewater treatment. It provides information on the challenges in the use of biochar for ARGs elimination in environmental and aqueous media. It also raises important gaps for future research. This review show that biochar can prevent antibiotic resistant through two processes (a) removal of co-selection triggers (contaminants) and (b) control of replication and transfer of ARGs. The results from this review indicate that co-composted biochar, biochar amended soils, and biochar amended wastewater treatment, will have lesser ARGs and bioavailable contaminants hence a reduced AR in non-clinical samples.

### 1.1 Sorption of contaminants by biochar

**Figure 3:**
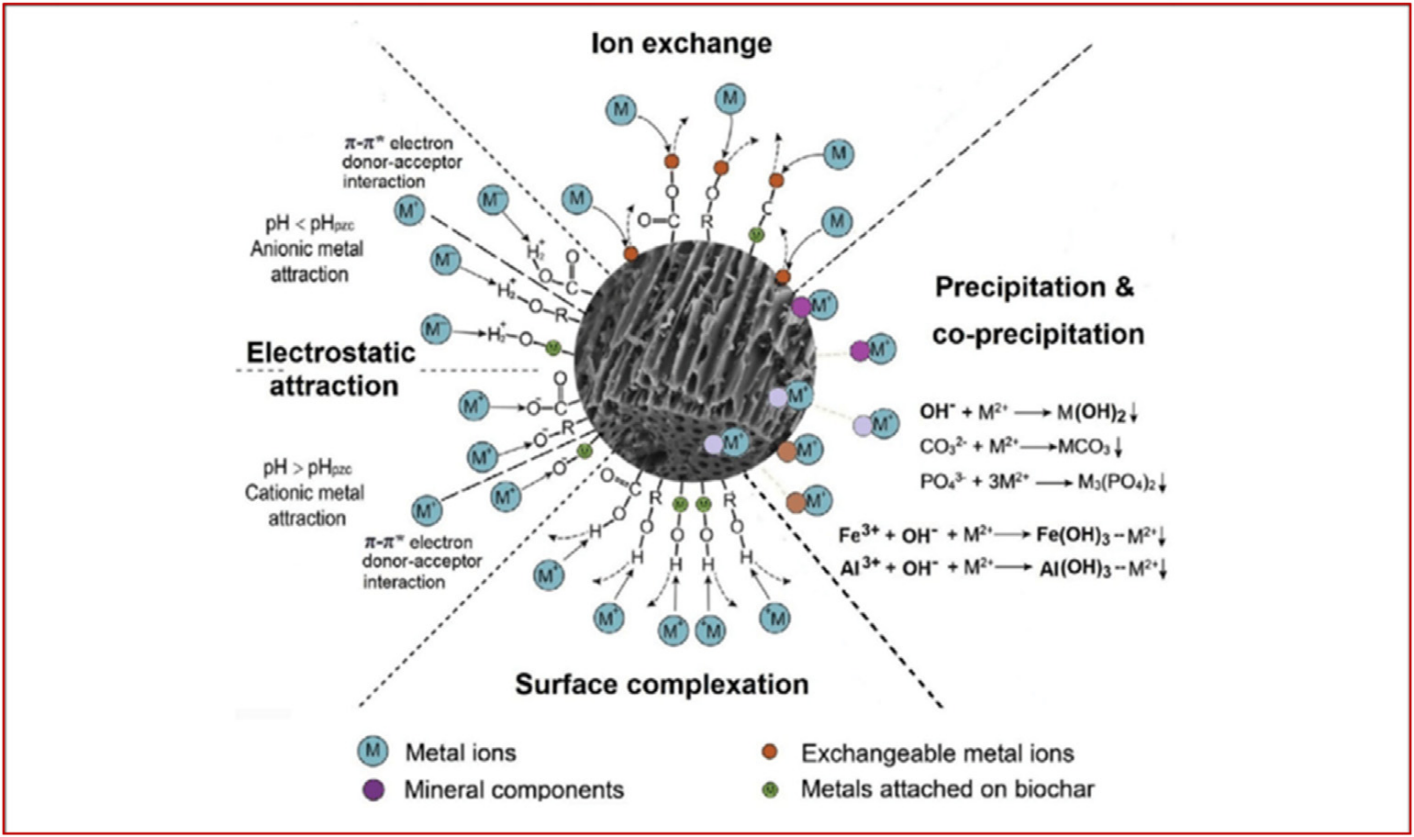
Mechanisms of metal sorption and stabilization in biochar (Source: Guo et al., 2020a).

**Figure 4:**
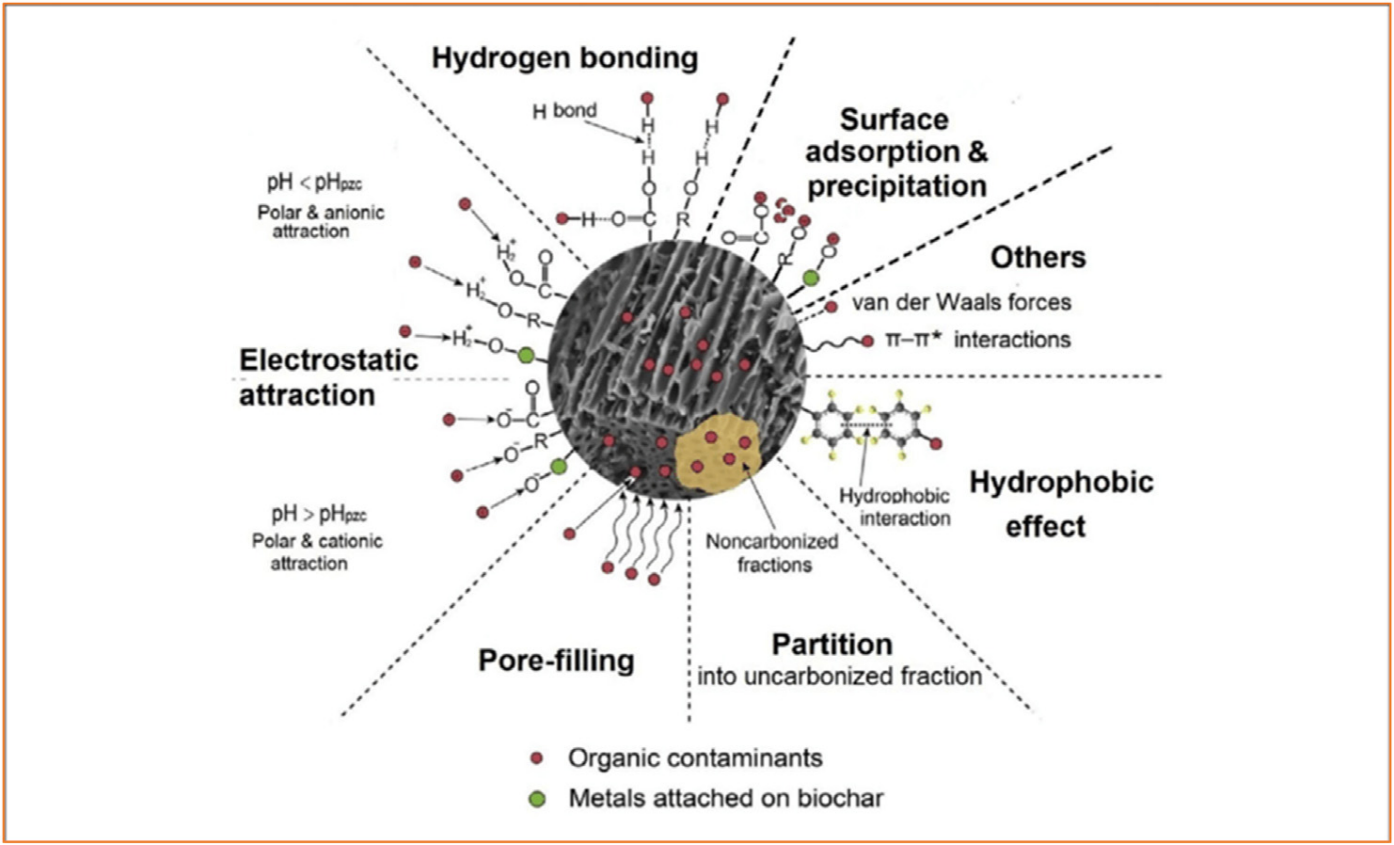
Mechanisms of organic contaminant sorption and stabilization in biochar (Source: Guo et al., 2020a).

Biochar have been reported in literature as effective amendment for the removal of contaminants in the environment including HMs (Dike et al., 2021; Hu et al., 2020; Liu et al., 2022a; Semple et al., 2013; Shakoor et al., 2020). Biochar is used to improve soil health and fertility due to its soil-conditioning effect and contaminant removal (Ahmad et al., 2014). Biochar reduces the bioavailability of PAHs and potential toxic elements (PTEs) in composting (Ye et al., 2019; Song et al., 2021) and wastewater treatment (Xu et al., 2021; Roha et al., 2021) through sorption, sequestration, and passivation. Biochar efficiently removed antibiotics in water (Ai et al., 2020; Hoslett et al., 2021), wastewater (Acelas et al., 2021; Ashworth and Ibekwe, 2020), soil (Ahmadabadi et al., 2019; Al-Wabel et al., 2021), and animal manure composting (Shan et al., 2018; Wang et al., 2016). Biochar can sorb HMs and antibiotics simultaneously (Cuprys et al., 2021) and can degrade sorbed antibiotics (Cui et al., 2022). The property of a biochar depends primarily on the feedstock, pyrolysis temperature, retention time, and heating rate. The behaviour of a biochar as amendment for contaminant removal depends on its properties and current state of aging/weathering (Semple et al., 2013). Biochar reduces PAHs extractability, bioaccessibility, bioavailability, and toxicity in soils and these effects increase with rise in biochar application rate (Bielska et al., 2017; Jimenez et al., 2018; Ogbonnaya et al., 2016). Biochar reduced water soluble fraction of phenanthrene hence indicating biochar as a suitable amendment to prevent PAHs leaching and bioaccessibility (Ogbonnaya et al., 2014; Jimenez et al., 2018). Adsorption of PAHs to biochar may depend on PAHs structure and molecular volume through pore filling and hydrophobic effect (Guo et al., 2017). It also depend on the properties of the biochar which is a factor of the feedstock and pyrolysis conditions (Jimenez et al., 2018). Biochar improved soil fertility and reduced bioavailability of PAHs in soil (Brennan et al., 2014). At 2–10% addition, biochar produced at low and high temperature, significantly reduced the bioaccumulation, dissipation, and bioaccessibility of PTEs and PAHs (Khan et al., 2015; Ni et al., 2017; Oleszczuk et al., 2017). Figures 3 and 4 shows the mechanisms of metal and organic contaminants sorption and stabilization by biochar.

## 2 Compost and biochar production

### 2.1 Composting

Composting is an aerobic decomposition process aimed at converting biowastes into useful, stable, high quality product with reduction in pathogens and environmental pollution (Bernal et al., 2017; Wichuk and McCartney, 2010). Composting recovers valuable organic matter and nutrient from biowastes. It reduces waste volume, odour, density, and moisture; eliminates toxic substances, pathogens, fly eggs and weed seeds (Bernal et al., 2017; Augustin and Rahman 2016). About 65% reduction in biowaste density is achieved after composting (Augustin and Rahman, 2016). Microorganisms (bacteria, actinomycetes, fungi), nematodes and arthropods contribute in composting through biodegradation and improvement of porosity and organic matter. Fungi thrive more at lower moisture (<35%) and don't do well at high temperature (around 60 °C). Bacteria and actinobacteria (actinomycetes) dominate in the thermophilic phase of composting, degrading lignin, cellulose, and hemicellulose while bacteria and fungi dominate at mesophilic and curing/maturation phase (Bernal et al., 2017). Conditions are optimized in active composting by managing aeration, porosity, moisture content, and composition of start materials. The use of earthworms (vermicomposting) can improve organic matter content, total nitrogen, and concentration of available nutrient in compost but may not be hygienic due to lack of proper thermophilic phase (Kästner and Miltner, 2016; Tognetti et al., 2007).

#### 2.1.1 Emissions

Gaseous emissions are monitored in composting to prevent air pollution and excessive loss of nitrogen. The gases of interest are mainly NH_3_, CH_4_, N_2_O, CO_2_, and H_2_S which are odorous and part of greenhouse gases (GHG) (Ren et al., 2020; He et al., 2020; Tian et al., 2020). High feedstock density, poor aeration, and reduced temperature may lower NH_3_ emission by generating anaerobic condition. This causes formation of organic acid leading to low pH, which can cause a shift from NH_3_ to ammonium. Around pH of 5.5–8.0 is optimum for composting though at high pH (>7.5), N may be lost through NH_3_ volatilisation (Wichuk and McCartney, 2010). Bulking agents which are mainly plant materials increase porosity (aeration) in composting. The density of the bulking agent is important as it contributes to its effect on composting aeration. A straw amended diary compost lost twice more N than a denser sawdust amended diary compost showing the effect of material density and porosity on N loss (Michel et al., 2004; Webb et al., 2012). Plant materials contain lignin and the degradation rate of biowastes in composting varies inversely with the lignin content. Fats and polyphenols can impede degradation during composting. This is evidenced in wastes from olive oil and wine industries. Mixing with fast degrading waste like biosolids and animal manure improve the composting process of these resistant materials.

Water content between 50% to 60% is best for composting and can drop between 45% to 35% during maturation. Less water content causes decrease in decomposition and high-water content causes anaerobic condition. Anaerobic conditions favour methanogenesis hence high moisture content and high temperature can be a contributing factor for CH_4_ generation. Increased porosity may reduce N_2_O, odour and CH_4_ emissions but increase NH_3_; high density may increase N_2_O and CH_4_ emission but reduce NH_3_; high temperature may support NH_3_ and CH_4_ emission but limit N_2_O (Bernal et al., 2017; Pardo et al., 2015). So, each decision on emission mitigation should be taken carefully to avoid pollution swapping. CO_2_ and N_2_O are released mainly at thermophilic phase and CH_4_ is released in anaerobic condition. CO_2_ is released through biowaste mineralization and respiration while N_2_O is formed from breakdown of biowastes rich in nitrogen. Release of odorous gases (H_2_S, NH_3_, VFAs, VSCs) is caused by type of compost materials (low C:N ratio, protein-rich waste) and composting condition (anaerobic: poor aeration, high moisture) (Tian et al., 2020). Adequate aeration (mainly at thermophilic phase), type of feedstock, and ratio of bulking agent to feedstock is important in reducing odorous gaseous emissions. The higher the porosity of the bulking agent the better the reduction in odorous gaseous emission though high porosity can cause NH_3_ emission. Chicken waste was composted with dry grass and straw at different aeration levels to monitor gaseous emission (NH_3_, N_2_O, CH_4_) and it was found that aeration reduced the emission of N_2_O and CH_4_ (Shen et al., 2011). According to Zhang et al. (2021c) C and N losses is caused highly by CO_2_ and NH_3_ emissions with little contribution from CH_4_ and N_2_O emissions.

#### 2.1.2 Feedstock C:N ratio

The C:N ratio and nutrient content of the composting feedstock determines the effectiveness of the composting and the C:N ratio (nutrient content) of the final compost. Animal waste and biosolid wastes usually contain low C:N ratio while plant wastes usually contain high C:N ratio. Breakdown of organic matter leads to reduction in C:N ratio of the compost to a desirable level. But at reduced C:N ratio, nitrogen loss can take place (mainly at high temperature and high pH) and reduce the quality of the compost through unwanted gaseous emissions (Bernal et al., 2017). Materials with low C:N ratio (e.g. livestock waste and sewage sludge) are mainly mixed with material of high C:N ratio (e.g. plant materials) for effective composting. Proper measurement of feedstock is essential in biowastes co-composting, to obtain required nutrient level at the end of the composting process. The quantity of feedstock to achieve a required starting C:N ratio is calculated from the C:N ratios of the various materials (Augustin and Rahman 2016). Plant wastes are used as bulking agent to increase porosity, humic acid, and lignocellulose in composting. This practice is important to achieve high compost mass and increase carbon content. A low feedstock C:N ratio (≤20:1) will lead to N losses while a high C:N ratio (≥40:1) can immobilise N and limit composting. Table 1 shows some organic wastes and their C:N ratio. Feedstock C:N ratio and moisture is responsible for N losses while porosity, aeration, and composting duration are responsible for C loses (Zhang et al., 2021c). C:N ratio of 25–40 is recommended for effective composting, though it may be desirable to increase this to 40–50 to mitigate N losses depending on the end use of the compost (Augustin and Rahman, 2016; Bernal et al., 2017). Starting C:N ratio of 50:1 lost below 10% N while 25:1 lost around 32% N (Michel et al., 2004). Animal wastes can trap essential nutrients during composting and act as a slow release nutrient source. Hen manure - straw compost and Pig manure - straw compost sequestered phosphorus as struvite (Hu et al., 2011).

**Table 1:** C:N ratio of commonly used materials for composting.

Material	C:N ratio	References
Cattle manure	17:1–19:1	Augustin and Rahman (2016); Klickitat compost Mix calculator
Cattle carcass	10:1	Augustin and Rahman (2016)
Corn silage	40:1	Augustin and Rahman (2016)
Corn stalk	68:1	Augustin and Rahman (2016)
Dairy manure	20:1	Augustin and Rahman (2016)
Grass clippings	17:1	Augustin and Rahman (2016)
Horse Manure	27:1–30:1	Augustin and Rahman (2016); Klickitat compost Mix calculator
Coffee grounds	14:1	Klickitat compost Mix calculator
Poultry carcass	4:1	Augustin and Rahman (2016)
Sawdust	442:1–466:1	Augustin and Rahman (2016); Bernal et al. (2017); Klickitat compost Mix calculator
Olive leaves	33:1–49:1	Bernal et al. (2017)
Sweet Sorghum bagasse	96:1	Klickitat compost Mix calculator
Garden pruning	21:1–186:1	Bernal et al. (2017); Klickitat compost Mix calculator
Poultry manure	6:1–10:1	Klickitat compost Mix calculator
Fresh leaves	37:1	Klickitat compost Mix calculator
Nut shells	35:1	Klickitat compost Mix calculator
Corn stalk	60:1–73:1	Klickitat compost Mix calculator
Alfalfa	12:1	Klickitat compost Mix calculator
Fresh leaves	37:1	Klickitat compost Mix calculator
Loose-dry and loose-wet leaves	47:1	Klickitat compost Mix calculator
Fruit waste	25:1–49:1	Klickitat compost Mix calculator
Cardboard	378:1	Klickitat compost Mix calculator
Food waste	15:1	Klickitat compost Mix calculator
Vegetable waste	11:1	Klickitat compost Mix calculator
Fresh weed	20:1	Klickitat compost Mix calculator
Grass (loose & compacted)	15:1	Klickitat compost Mix calculator
Newsprints	54:1	Klickitat compost Mix calculator
Wood chips	226:1–600:1	Augustin and Rahman (2016); Bernal et al. (2017); Klickitat compost Mix calculator
Cotton gin waste	51:1	Klickitat compost Mix calculator
Rice straw	76:1	Bernal et al. (2017)
Maize straw	62:1	Bernal et al. (2017)
Wheat straw	53:1–60:1	Bernal et al. (2017); Klickitat compost Mix calculator
Citrus pruning	24:1	Bernal et al. (2017)
Palm tree pruning	15:1–190:1	Bernal et al. (2017)
Sheep manure	14:1–16:1	Augustin and Rahman (2016); Klickitat compost Mix calculator
Swine carcass	14:1	Augustin and Rahman (2016)
Swine manure	12:1–13:1	Augustin and Rahman (2016); Klickitat compost Mix calculator
Turkey litter	16:1	Augustin and Rahman (2016)

#### 2.1.3 Stability and maturity

Stability of a compost refers to level of organic matter decomposition while maturity refers to non-adverse effect level of the compost (Wichuk and McCartney, 2010). A stable compost is that which have reached a high level of decomposition with resistance to further decomposition. This is normally determined using microbial activity indices. A mature compost is one which do not cause any adverse effect when applied to soil. It is mainly determined using plant bioassay studies. The stability of a compost is a major parameter in enhancing PAHs biodegradation when compared with soil-to-compost ratio and pollutant concentration (Sayara et al., 2010). Compost stability and maturity are mostly used interchangeably. They are measured using physical, chemical, and biological indices which include temperature, pH, C:N ratio, humification, colour and odour, respiration (O_2_ demand and CO_2_ evolution), organic matter, phytotoxicity, cation exchange capacity, electrical conductivity, dissolved organic carbon, enzyme activity, nitrate:ammonium ratio, and spectroscopy (Wichuk and McCartney, 2010).

No single test is enough to measure stability and maturity; a combination of tests is usually used. Most test methods are compared with respirometry result to arrive at conclusion and the acceptable test for compost stability and maturity varies among nations. Return to ambient temperature after thermophilic phase, stable C:N ratio over time, stable O_2_ intake (or CO_2_ release over time), reduced odour, reduced toxicity/mutagenicity, dark/humus colour, amongst others are used to determine compost stability and maturity (Tognetti et al., 2007; Wichuk and McCartney, 2010). The oxygen demand and carbon dioxide evolution must be within recommended standard of the country or region if it is used for stability and maturity testing. Self-heating or re-heating should be checked if using temperature for stability. The standard C:N ratio of ≤25 should be attained before subjecting the compost to any stability and maturity testing and rating (Wichuk and McCartney, 2010). According to Guo et al., in 2020, compost maturity can also be estimated using the T-value (which is a measure of the ratio of final C:N ratio to initial C:N ratio). T-value of ≤0.6 can indicate the level of maturity of the compost (Guo et al., 2020c). This can be interpreted as the lower the T-value below 0.6, the better the compost maturity and vice versa.

#### 2.1.4 Composting and ARGs

The use of antibiotics in animal management increases AR and ARGs expression in bacteria and animal manure compost. This poses a human health risk because the ARGs are not efficiently removed by composting (Yoshizawa et al., 2020). Microbes isolated from food waste compost were found to possess AR and the use of such compost is not safe for humans and the environment (Furukawa et al., 2018). Composting failed to reduce the abundance of sul1 (which is responsible for sulphonamide resistance) but reduced sul2 and intl1 in *Escherichia coli* (Lin et al., 2021). Increased abundance of ARGs was observed after composting of pig manure which was attributed to the microbial structure in the compost (Cao et al., 2020b). Backyard compost was found to contain less ARGs than commercial compost and ARGs in the commercial compost, indicated a high risk of microbial inhalation than ingestion in compost application due to the microbes associated with the ARGs (Mao et al., 2021). This is due to the high content of antimicrobials in the feedstocks used for commercial compost.

Composting of digestate reduced the level of ARGs but a co-composting with fresh feedstock impeded ARGs removal (Gurmessa et al., 2021). Anaerobic digestion has reduced the ARGs which enhanced its removal through composting but the addition of fresh feedstock introduced more ARGs and ARB. Aerobic composting and anaerobic digestion was reported to reduce copies of ARGs in dairy Cow manure but anaerobic digestion was more effective (Katada et al., 2021). Despite the reduction in copy numbers observed in the treatments, several ARGs copies still remained, and aerobic composting was not effective against tetA and tetB genes. The temperature in anaerobic digestion may be a contributory factor in its increased effect on ARGs. It was recommended that thermophilic composting may be more efficient in reducing ARGs in compost than conventional composting technique (Sardar et al., 2021). There was a decrease in ARGs and MGEs in sewage sludge composting through the use of a semi-permeable membrane in the thermophilic phase (Cui et al., 2020a). ARGs and MGEs increase was observed in a conventional thermophilic phase without any membrane and atmospheric microbial contamination was postulated to be responsible. Industrial composting of sewage sludge reduced ARGs through reduction of the population of ARB (Lopez-Gonzalez et al., 2021). Bulking agents were implicated in the abundance of ARB in composting in a study using corn stalk. The use of pesticides and herbicides in farming contributes to the expression of ARGs in microbes found in such bulking agents (Su et al., 2015). Though composting is not very effective in removal of ARGs from animal manure, the abundance of ARB is lower in a composted swine, cow, and chicken manure than the fresh manure (Wang et al., 2019a). From the information above, it can be deduced that anaerobic digestion is better than composting for reduction of ARGs. However, composted manure is safer than a fresh manure as composting can still reduce ARGs abundance even though it is not effective.

### 2.2 Biochar production and properties

Biochar is a carbon rich product produced by biomass pyrolysis with little or no air/oxygen (Ahmad et al., 2014). The type of feedstock used in biochar production, pyrolysis temperature, retention time, and rate of heating, affects the biochar properties (Uchimiya et al., 2011; Liu et al., 2018a, Liu et al., 2018b; Tomczyk et al., 2020). Solid wastes and animal litter gives a high biochar yield than crop residue and wood biomass. This can be seen in the high ash content of these feedstocks (animal litter and solid waste) due to high inorganic content. But plant waste feedstock generates biochar of higher surface area and carbon content than animal wastes due to difference in lignin and cellulose content (Tomczyk et al., 2020). From Yin et al. (2021) biochar produced at 500–900 °C was better for preventing CH_4_ and N_2_O emissions while those produced at 200–500 °C were better for reducing NH_3_ emission in composting. Crop remains and wood biomass derived biochar were better for reducing gaseous emissions in composting. This buttresses the effect of feedstock and pyrolysis temperature on biochar properties.

Temperature is an important factor in biochar production as increase in temperature improved biochar quality for environmental application (Xia et al., 2010; Zimmerman, 2010; Zhang et al., 2020). High temperature (HT) formed biochar are more stable, better sorbents, and less susceptible to weathering than low temperature (LT) formed biochar (Xia et al., 2010; Zimmerman, 2010; Zhang et al., 2020). Higher pyrolysis temperature (500–1000 °C) increase pH, surface area, ash content, carbon content, electrical conductivity, porosity (by increase in pore size), and aromatic/aliphatic ratio of biochar while it reduces O/C ratio, H/C ratio, DOC, –COOH and –OH functional groups (Uchimiya et al., 2011; Liu et al., 2018a, Liu et al., 2018b; Zhang et al., 2020). This improves the sorption efficiency and stability of the biochar. At high temperature, some of the organic functional groups from the biochar feedstock are lost thereby creating more space for sorption efficiency in the biochar. The efficacy of biochar use in contaminants remediation depends on its surface area, size of the contaminant, porosity, and the functional groups on the surface of biochar (Enaime et al., 2020). LT biochar have small pore size, low surface area, and high O-containing surface functional groups which make it suitable for the sorption of inorganic compounds, while HT-biochar have higher surface area, higher micropore volume, and hydrophobic property making it suitable for the sorption of organic compounds (Enaime et al., 2020). HT-biochar (around 900 °C) and small particle sized biochar (<2mm) have higher and better contaminant sorption efficiency (Jin et al., 2022). This can be attributed to surface area, ash content, and porosity as biochar with high surface area and porosity was found to have a better sorption effect (Lou et al., 2011).

Kimbell et al. (2018) stated that the conversion of biosolids to biochar can remove/reduce ARGs depending on the pyrolysis temperature and retention time. Pyrolysis at the range of 300–700 °C reduced ARGs in municipal biosolids and at temperature of ≥500C, ARGs was significantly decreased (Wang et al., 2021a). Batch pyrolysis at 500 °C for 5 min, reduced the ARGs below detectable limits. Conversion of dewatered swine manure to biochar greatly lowered the ARGs and the use of the biochar in anaerobic digestion prevented HGT by 74.8% through mitigating abundance of MGEs, indicating its effectiveness in managing ARGs (Wang et al., 2022).

Formation of biochar at HT help reduce binding of contaminants to the biochar during production as the use of biochar that contain contaminants can cause environmental contamination (Freddo et al., 2012; Godlewska et al., 2021). This assertion is most likely the case for organic contaminants and not PTEs. Conversion of feedstocks to biochar through pyrolysis decreased the level of various contaminants (dioxins, PAHs, PCBs, antibiotics, ARGs, microplastics, antimicrobials, per- and polyfluoroalkyl substances) with great effectiveness between 95% - 99% (Buss, 2021). The organic contaminants were reduced below detectable limits. Conversion of sewage sludge to biochar reduced the PAHs concentration (Zielinska and Oleszczuk, 2016). Pyrolysis at >600 °C (for 2 h) removed PCBs, PAHs, and pharmaceutical contaminants from sewage sludge (Mosko et al., 2021). Discrepancies exist in literature on the factors influencing contaminant yield in biochar during production. Biochar feedstock, type of reactor, and production temperature are the most important. The yield of PAHs in biochar has no significant dependence on pyrolysis temperature, but its distribution and speciation changes with temperature as high molecular weight PAHs was found to increase in biochar with increase in production temperature (Adanez-Rubio et al., 2021). PTEs appears to increase with rise in temperature irrespective of other conditions while the yield of PAHs is affected by all production conditions (Keiluweit et al., 2012). The content of PTEs in sewage sludge-derived biochar increased with increase in pyrolysis temperature (Kong et al., 2019) and the conversion of sewage sludge to biochar reduced PAHs content but increased PTEs (Zielinska and Oleszczuk, 2015). A biochar produced by fast pyrolysis had less PAHs concentration, and biochar produced using rotary reactors had less PAHs than those from batch reactors (De la Rosa et al., 2016; De la Rosa et al., 2019). The PAHs yield in the batch reactor biochar reduced with increase in temperature of pyrolysis. However, earlier reports have stated that, slow pyrolysis derived biochar had lower PAHs and lower bioaccessible PAHs (which reduced with increase in production temperature and retention time) when compared to fast pyrolysis biochar (Fabbri et al., 2013; Hale et al., 2012). Based on these discrepancies, use of non-contaminated and less contaminated feedstock is best for biochar production as no production condition is perfect for complete elimination of contaminants from contaminated feedstock.

Duration of pyrolysis is a factor in the property of a biochar. Biochar yield reduces with increase in pyrolysis temperature (Chandra and Bhattacharya, 2019; Zhang et al., 2020; Das et al., 2021a). Increase in pyrolysis duration have similar effect on biochar quality like increase in pyrolysis temperature (Mia et al., 2015; Chandra and Bhattacharya, 2019). Both improves the stability and quality of biochar for application as environmental amendment. Therefore, biochar formed around 650 °C at longer heating duration, will be more suitable for amendment due to its high durability (stability and long half-life) and less labile organic matter (Semple et al., 2013; Das et al., 2021a). Pyrolysis temperature does not affect nitrogen content of biochar as this is dependent on type of feedstock (Ahmad et al., 2014). Hence, nitrogen rich wastes can be used as biochar to sorb contaminants and supply nitrogen in soils. Depending on the end use of the biochar, proper selection of feedstock and production at the right pyrolysis temperature is important, to produce biochar with the desired qualities for a given environmental application.

## 3 Biochar in biowastes composting

Biochar have been reported to enhance the effectiveness of biowastes composting and to improve the quality of the final product (Chavez-Garcia et al., 2020; Godlewska et al., 2017; Liu et al., 2017; Pandiyan et al., 2021; Teodoro et al., 2020; Wang and Zeng, 2018; Wu et al., 2017b; Xiao et al., 2017; Yin et al., 2021; Zhang et al., 2021c). Biochar has been shown in literature to reduce gaseous emissions (Awasthi et al., 2017, 2020a), reduce N and C losses (Awasthi et al., 2020b; Zhang et al., 2021a), reduce composting duration (Qu et al., 2020), increase humification (Vandecasteele et al., 2014; Jindo et al., 2016), improve aeration and porosity (Zhang et al., 2014), reduce bioavailable HMs fraction (Cui et al., 2020b), reduce ARGs (Qian et al., 2019; Cui et al., 2016a, 2016b), improve microbial activity (Awasthi et al., 2020b; Lia et al., 2020; Qu et al., 2020), improve available nutrient (Zainudin et al., 2020; Wei et al., 2021), and reduce ecotoxicity in compost (Wang et al., 2017).

Agyarko-Mintah et al. (2017a) found no significant difference in reduction of GHG emission and C/N losses (feedstock ratio; Poultry litter: Sugarcane straw 2:1) despite using biochar from difference sources. Though the green-waste biochar was better than the poultry litter biochar in reducing CH_4_ and N_2_O. Reduction in C and N losses were better in green-waste and poultry litter respectively and this can be attributed to the C:N ratio of the feedstocks. When a different feedstock ratio was used (Poultry litter: Sugarcane straw 5:1), green-waste biochar amended compost had higher N retention than poultry litter amended biochar and this is through preventing NH_3_ emission (Agyarko-Mintah et al., 2017b). This shows the effect of feedstock ratio on composting and compost quality. Layer hen manure and sawdust co-composting was amended with biochar from different feedstocks (cornstalk, woody, bamboo, layer hen manure, and coir) and there was reduction in N losses, CH_4_ and NH_3_ emissions in all treatment (Chen et al., 2017). The surface area, pore volume, and C:N ratio of biochar appear to be a factor for emission and N losses reduction with the first two having greater effect. Reduction in N losses was dependent on surface area and pore volume irrespective of C:N ratio. Biochar reduced N losses and improved N availability in N-limited wastes. N losses decreased in Olive mill waste and sheep manure compost (López-Cano et al., 2016). Biochar improved maturity, reduced N losses, and eliminated phytotoxicity in composting of beer vanasse (Wang et al., 2017). Poultry manure and cow manure were co-composted with apple pomace, rice straw, and rice bran together with biochar amendment (Jindo et al., 2016). Biochar amended compost had improved humic acid (HA) and fulvic acid (FA) with enhanced stability. The effect of biochar on gaseous emissions and quality of compost have been greatly reviewed and more information can be found in the references in the first paragraph of this heading.

## 4 Effect of biochar on ARGs in composting

Composting of biosolids and animal wastes is a waste management strategy aimed at reducing environmental contamination and improving manure quality. Biochar application in composting has been proven effective in improving compost quality. However, little is known on the impact of composting and biochar co-composting on ARGs which can exist in these wastes through the use of antibiotics by humans and in animal management.

### 4.1 Biochar, composting feedstock, and microbial community

Biochar enhanced ARGs removal in anaerobic digestion by altering intI1 gene (Yang et al., 2021b). Biochar inhibited the lateral transfer of ARGs carrying plasmids in *Escherichia coli* and this effect improved with increase in biochar pyrolysis temperature (Fang et al., 2022). Biochar can attenuate *Escherichia coli* preventing possible ARGs uptake. Biochar can also adsorb plasmids and the sorption efficiency is enhanced with elevation in biochar pyrolysis temperature. Biochar size have an impact on the effect of biochar on ARGs. Although ARGs can sorb to bulk biochar, nano-sized biochar were reported to remove ARGs and extracellular genetic materials through both sorption and fragmentation (Lian et al., 2020). The nano-biochar repressed genetic replication and damaged the genetic materials through release of toxic free radicals. Addition of biochar to pig manure significantly reduced the ARGs but the application of biochar to poultry litter did not affect/reduce the prevalence of Class 1 Integron which indicates their recalcitrance and biochar inefficiency in managing it (Mohammadi-Aragh and Stokes, 2021). Biochar effectiveness in mitigating ARGs during composting may be dependent on the type of composting feedstock which in turn determines the bacterial community, dominant bacterial phylum, and possible ARGs host. Therefore, the properties of the biowastes and its indigenous microbes can affect the efficiency of biochar in eliminating/mitigating ARGs. According to Cui et al., in 2017, the effect of biochar on ARGs during composting is dependent on the properties of the manure and that of the biochar (Cui et al., 2017). Though biochar-sorbed antibiotics can still be bioavailable (exerting pressure on the microbial community), HT-biochar could have the potential for effective sorption and ARGs inhibition (Wang et al., 2020).

The presence of certain bacterial groups appears to affect the removal and distribution on ARGs in composting. Proteobacteria, Actinobacteria, and Bacteroidetes have been suggested to play a role in ARGs dissipation (Li et al., 2019c, 2020). Rice straw biochar failed to reduce ARGs in chicken manure composting when compared to mushroom biochar and this was attributed to the presence of Actinobacteria and Firmicutes (which could distribute ARGs in the compost) and not the property of the biochar (Cui et al., 2016a). In the presence of hyperthermophiles, biochar addition enhanced ARGs elimination during municipal sludge composting, though this was not significant (Fu et al., 2021). Application of biochar resulted only in a 4% increase in ARGs removal when compared to hyperthermophiles (80.7%). Firmicutes and Nitrospirae were implicated in ARGs abundance as a possible host. Organic carbon, pH, and intI1 (class 1 integrase gene) had significant impact on ARGs control. **B**iochar declined ARGs in chicken manure composting and Firmicutes were implicated as the possible ARGs hosts (Zhou et al., 2021). The intI1 gene correlated with the ARGs and this can be distributed through HGT hence implicating HGT as a possible determinant of ARGs in composting. Biochar addition improved microbial diversity, significantly reduced ARGs, and completely removed tetracycline during sewage sludge vermicomposting, in a dose dependent manner (Huang et al., 2020). Inhibition of ARGs was found to be dependent on the impact of biochar on Firmicutes and Proteobacteria and this effect was dose dependent (Sun et al., 2018).

Aeration and dissolved organic matter (DOM) affect ARGS removal and microbial community in biochar-amended composting (Feng et al., 2021). Improved aeration and degradation of DOM enhance ARGs dissipation (Li et al., 2019c, 2020). Humic-acid, heavy metals, and dissolved biochar affects the transfer of ARGs in bacteria (Liu et al., 2021b, 2022b). Interaction of metal ions with HA or dissolved biochar can repress or promote ARGs transfer. HA can passivate metals preventing their bioavailability and may promote metal bioavailability by desorbing biochar-sorbed metals. Understanding the interaction of metal ions with HA and dissolved biochar can help in managing ARGs in soils and composting through proper manipulation of their concentrations. The reported effect of organic carbon, DOM, and HA on ARGs is similar to the effect of clay. Clay reduced ARGs abundance and distribution in poultry manure composting and this effect is proportional to dose (Awasthi et al., 2019a, 2019b). These reports seemingly indicate that, the parameters which affect contaminants bioavailability can also affect ARGs abundance and distribution.

### 4.2 Impact of HMs

Presence of HMs can influence biochar effect on ARGs and this effect is more dependent on the bioavailable fraction (BF) of the metals (Cui et al., 2016a). A decrease in the BF of the metals will enhance biochar effect on ARGs. Biochar use in chicken manure composting declined ARGs by reducing the relative abundance of the ARGs and BF of metals hence preventing co-selection effect (Li et al., 2017). Temperature was reported as the most influencing environmental parameter controlling ARGs abundance and distribution during Chicken manure composting followed by C:N ratio and pH. Biochar reduced the abundance of MGEs and ARGs in pig manure composting with no effect on copper resistance genes, indicating that removal of co-selection pressure may not always be a factor in all ARGs reduction in composting (Qian et al., 2019). Variation and shift in microbial community/structure explained the changes observed in MGEs and ARGs in the study. A similar study in 2018, indicated that ARGs behaviour in biochar amended vegetated soil was related with antibiotics and not bioavailable HMs fraction (Cui et al., 2018). The ARGs behaviour associated stronger with MGEs and available phosphorus which could indicate the influence of nutrient on microbial diversity, proliferation, and ARGs distribution. Biochar was found to reduce ARGs and metals in sewage sludge compost with Firmicutes as possible host for ARGs (Qiu et al., 2021b). The metal concentration had positive correlation with sul1 and intI1; the intI1 gene, HMs and bacterial community were major contributors to the ARGs patterns observed. Coconut shell biochar at 7.5% dose, was able to reduce HMR bacteria abundance in poultry manure composting through altering the interaction between the dominant bacteria community (potential ARGs host) with important environmental parameters (Awasthi et al., 2021). This affects bacteria and ARGs distribution and hence reduces the abundance of ARGs.

The proper dose for effective HMs passivation, ARGs and MGEs elimination in composting is not yet understood. A range of 2–10% application dose have been reported to be effective in the reduction of HMs bioavailability and ARGs abundance. An addition of 5% dose of biochar had no better effect over 2% (Fu et al., 2021); 10% was proposed as the best over 5% and 20% (Li et al., 2017); 7.5% was better than 2.5%, 5%, and 10% (Awasthi et al., 2021); and 6% was proposed as the best over 12% and 24% (Wang et al., 2018). There is a concern that biochar at 12% dose may increase the distribution and transfer of ARGs (Wang et al., 2018). A biochar dose of 5–10% was reported by Akdeniz et al. (2019) to be potentially effective in improving the quality of animal waste composting. Table 2 shows some biowastes composting, some biochar production conditions, biochar application dose, and biochar effects on ARGs.

**Table 2:** Some composting parameters and biochar effect on ARGs.

Biochar feedstock	Compost feedstock	Biochar dose (%)	Pyrolysis temperature (^o^C)	Pyrolysis duration (hours)	Composting duration (days)	Effect of biochar application on ARGs in composting	References
Rice straw & mushroom	Chicken manure & sawdust; 3:2 v/v	5 dwt	NA	NA	42	0.86 log unit reduction of ARGs. Mushroom biochar had positive effect on ARGs elimination while rice straw biochar was the opposite. Bioavailable metal fraction affects ARGs removal	Cui et al., 2016a, Cui et al., 2016b
Rice straw & Mushroom residue	Pig manure & sawdust; Duck manure & sawdust	5 dwt	500	4	42	Mushroom biochar removed ARGs in pig manure while rice straw biochar was the opposite. Both biochar negatively affected ARGs removal in duck manure Mushroom biochar reduced metal total & bioavailable fraction	Cui et al. (2017)
Tree leaves	Sludge, back-mix peanut shell; 3:1:1 w/w/w	2 & 5	550	3	66	Biochar addition had a 4% increase in ARGs elimination when compared to hyperthermophiles (80.7%). There was no significant different between the two biochar doses (2% and 5%)	Fu et al. (2021)
Corn cob & rice husk	Sludge & *Eisenia fetida*	1.25 & 5	NA	NA	60	ermF & tetX genes were decreased by corncob biochar. Sul1 & sul2 were decreased by rice husk biochar at 5% dose. Intl1 increased with increase in biochar concentration.	Huang et al. (2020)
Bamboo	Chicken manure & wheat straw; 1:1 w/w	5, 10, 20 dwt	600	NA	28	All biochar dose had no effect on tetG, ​tetW, ​tetX, ​sul2, ​drfA1, & ​ermB. 10% biochar was effective against tetC & drfA7. **B**iochar decreased bioavailable metal concentration.	Li et al. (2017)
Apple tree branches	Pig manure & wheat straw; 5:3 w/w	10 dwt	550	NA	40	Biochar addition reduced ARGs (tetC, ​tetG, ​tetQ, ​tetX, ​sul1, and ​ermB) by 0.23–1.09 logs, MGEs (intI1 & ISCR1) by 26–85%, and intI2 ​& Tn914/1545.	Qian et al. (2019)
Maize straw	Sewage sludge & maize straw 3:2 v/v	5	400	8	40	Biochar reduced ARGs by 17.6%. Actinobacteria, Firmicutes, Bacteroidetes, and Proteobacteria were dominant with Firmicutes as possible ARGs host.	Qiu et al. (2021b)
Sawdust, corn Stover & peanut hull	Swine manure & corn stover 8:1 w/w	6, 12, 24	600	NA	30	6% biochar dose enhanced ARGs elimination. 12% dose and above, may increase ARGs distribution and abundance.	Wang et al. (2018)
Maize straw	Fresh chicken manure & mushroom residue 1:1 w/w	5	400	8	42	Biochar addition was 6.1% better in ARGs removal than control. Firmicutes implicated as possible hosts for ARGs. HGT responsible for ARGs distribution through intI1.	Zhou et al. (2021)
Coconut shell & Bamboo	Pig manure & wheat straw (5:1 dwt)	10	NA	NA	42	Firmicutes, Actinobacteria, Proteobacteria, and Bacteroidota as dominant ARB groups Biochar resulted in 40%–60% ARB reduction.	Awasthi et al. (2022)

## 5 Biochar on ARGs in wastewater

In addition to the effect of biochar on HMs, biochar have also been implicated in the control of ARGs in wastewater treatment plants (WWTPs) (Barancheshme and Munir, 2018). Use of biochar in anaerobic digestion of cattle farm wastewater significantly reduced the ARGs by reducing the relative abundance of the genes through influencing the relative abundance of possible bacterial hosts (Firmicutes and Proteobacteria) (Sun et al., 2018). Beta-cyclodextrin functionalised biochar was effective in the reduction of ARGs in HMs and dye co-contaminated wastewater (Wu et al., 2020). Alteration of microbial diversity which led to a reduction in possible ARB was hypothesized as a reason for the ARGs reduction. In a recent report by Bimova et al., biochar was able to remove 90% pharmaceuticals, 99% RNA, and 100% of DNA from wastewater (Bimova et al., 2021). The report indicates that biochar can prevent co-selection pressure from contaminants in wastewater and can prevent HGT through destruction and removal of any available genetic material.

## 6 Effect of biochar on ARGs in soils

Conversion of biowastes to biochar reduced ARGs, MGEs, and increased the HMs concentration in the biochar but these HMs lacked bioavailability, mobility, and migration potency (Lin et al., 2020; Zhou et al., 2019). Use of this biowaste biochar (at 2% dose) as soil amendment greatly reduced HMs bioavailability in soil, accumulation in plants, and removed ARGs to background level. Biochar was able to decrease the phyllosphere ARGs in class 1 integron gene cassette pool in a soil amended with struvite (An et al., 2018). Most of the genes were coding for resistance to beta-lactam antibiotics, aminoglycosides, and chloramphenicol. Biochar significantly reduced ARGs in non-vegetated soil but had no significant effect on ARGs in rhizosphere and phyllosphere, indicating plants to affect ARGs in soils (Chen et al., 2018b). Plants affected microbial community structure, which affected the efficacy of biochar on ARGs in the amended soils, hence biochar alone may not be effective in reducing ARGs in some vegetated soils. Use of 2% biochar, reduced oxytetracycline ARGs in soil, leaves, and root of plant and this is attributed to the reduction of Firmicutes (Duan et al., 2017). However, it was found from a two years laboratory study, that biochar can maintain soil microbial diversity, and high soil moisture content enhances ARGs dissipation (He et al., 2021). The high moisture could lead to leaching of ARGs in soil while the maintained microbial structure could improve propagation of ARGs through HGT.

Application of biochar to soil reduced the ARGs which was influenced by BF of Cu (Li et al., 2020). This shows the influence of HMs BF on co-selection pressure and AR. The bacteria in the groups Firmicutes and Actinobacteria were implicated as major potential contributors of soil ARGs. In a sulfamethazine amended soil, biochar promoted DOM transport in soil, repressed sulfamethazine vertical movement, suppressed sul2 gene but have no influence (and did not prevent vertical transfer of sul1) on sul1 gene (Qiu et al., 2021a). Sul2 gene behaviour was reported to be dependent on antibiotic concentration while sul1 gene was dependent on antibiotic concentration and DOM, implicating DOM in the leaching of ARGs in soils. Calcined eggshell enhanced the dissipation of antibiotics in soil, reduced antibiotic water soluble fraction, and repressed the diversity of ARB and ARGs in crop (Ye et al., 2016a). Co-application of biochar and pyroligneous acid improved soil properties, reduced ARGs bacteria host, repressed HGT, lessened metal bioavailability, and decreased ARGs (Zheng et al., 2021). Similarly, an earlier article reported that, application of biochar to soil enhanced the dissipation of antibiotic and greatly reduced ARGs in old and new leaves of plants (Ye et al., 2016b). It can be deduced from the research reports that biochar can reduce co-selection pressure and control ARGs in soils.

## 7 Biochar influence on PTEs contamination-related co-selection pressure

It has been established that metals affect the AR behaviour of bacteria in environmental media (Baker-Austin et al., 2006). A metal and an antibiotic may share same antimicrobial toxicity mechanism hence resistance against one cause resistance against the other. This fact has been highly reported and reviewed in literature (Maurya et al., 2020; Pal et al., 2017; Seiler and Berendonk, 2012). The presence of organic contaminants (e.g. PAHs, PCBs) can cause a shift in microbial structure which can lead to microbial community with high abundance of ARGs and HGT-capable bacteria (Gorovtsov et al., 2018). Among all contaminants that contribute to co-selection pressure, PTEs has been shown to have the greatest effect (Zhao et al., 2020; Mazhar et al., 2021). The effectiveness of biochar in the remediation of PTEs and organic contaminants in contaminated environmental media is richly represented in literature (Ho et al., 2017; Kumar and Bhattacharya, 2021; Mansoor et al., 2021; Murtaza et al., 2021; Wang et al., 2019b). Environmental remediation of PTEs and organic contaminants using biochar leads to reduction in mobility, bioavailability, bioaccessibility, and possible toxicity of the contaminants. Since biochar can reduce the bioavailability and bioaccessibility of contaminants, it can prevent possible co-selection pressure exerted by the contaminants, hence reducing the abundance of ARGs and subsequent AR.

### 7.1 Biochar effect on PTEs in composting

The application of biochar in composting can reduce the bioavailable and total metal concentration in compost, and the fate of ARGs in composting correlates better with bioavailable HMs fraction than total fraction (Cui et al., 2016b; Qiu et al., 2021b). Composting improves microbial abundance while the addition of biochar improves microbial diversity and reduce metal concentration in composting (Zhang et al., 2021b). Firmicutes seems to be the dominant bacterial phylum in biochar amended composting (Zhang et al., 2021b). Use of biochar and lime at 1:1 during composting of dewatered fresh sewage sludge, reduced the bioavailable concentration of Cu, Zn, Pb, and Ni which ranged from 34-87% reduction (Awasthi et al., 2016). In pig manure composting, 12% dose of wheat biochar and rice straw biochar immobilised Cu and Zn (Awasthi et al., 2020c). In similar studies, 10% biochar dose, passivated and reduced the BF of Zn, Cu, Cd, and Pb with a greater effect on Cu and Pb (Cui et al., 2020b; Duan et al., 2021; Hao et al., 2019; Li et al., 2017; Qian et al., 2019). The bacterial diversity was increased and altered at thermophilic phase, and the effect of bacterial on metal passivation was dependent of C:N ratio (Song et al., 2021). In another study, during poultry manure composting, biochar reduced the extractable fraction of HMs (Cu, Zn, As, and Cd) with a significant effect on Cd and As (Zhang et al., 2021a). Recent studies also reported the positive impact of biochar on microbial groups and HMs (Cu, Zn, Cr, Pb, Ni) passivation (Liu et al., 2021a; Song et al., 2021). The transformation of Zn and Cu to stable forms was reported to be influenced by HA and FA in compost (Li et al., 2019b; Song et al., 2021). Biochar could passivate and attenuate HMs in vermicomposting (Paul et al., 2020). Ye et al. (2019) reported that incorporation of biochar in soil composting remediation led to a decreased PAHs and PTEs concentration. The co-composting enhanced microbial activity and increased contaminant biodegradation, biotransformation, and sorption.

### 7.2 Biochar effect on PTEs in soil

The use of biochar in HMs (Pb, Zn, Cd, Cu) contaminated soils improves soil properties, promotes plant growth, and reduces the accumulation of the HMs in plants (Chen et al., 2018a; Wang et al., 2019c). In a study using Cd as HMs surrogate, the authors stated that biochar can adsorb HMs thereby reducing their bioavailability, mobility, and plant uptake (Ahmed et al., 2021). In a study using LT (350 °C) and HT (650 °C) pyrolyzed green waste, the LT-biochar had a better effect on reducing the BF of the HMs (Pb, Cr, Cd) which was attributed to functional (O-containing) groups and physicochemical properties of the LT-biochar (Aslam et al., 2017). This simply indicates that biochar pyrolysis temperature has effect on biochar-HMs interaction. In addition to reduction in HMs (Cd, Cu, Ni, Zn, Pb) concentrations, mobility, and bioavailability, biochar also improved soil properties and microbial activity in amended soil (Cui et al., 2013; Liu et al., 2018b; Lu et al., 2017; Yang et al., 2016). Biochar increased the residual fraction and reduced the exchangeable fraction of HMs (As, Zn, Pb, Cd, Cu) in a soil contaminated by mining activities (Dai et al., 2018; Dang et al., 2019). In addition to pyrolysis temperature, biochar particle size and source of feedstock affects biochar-metal interaction (Boni et al., 2021). A fine particle biochar was better than a coarse biochar in immobilizing and reducing extractable and leachable fractions of Cd and Pb (Fahmi et al., 2018). Biochar was applied to yellow and cinnamon soils and the biochar transformed the reducible and extractable fraction of the HMs (Cd, As) into the residual fraction, hence reducing mobility and bioavailability (Luo et al., 2020). The C/O, surface area, and total pore volume of the biochar affected the leachability and stabilization of the HMs while the high surface area, increased porosity, and organic matter of biochar improved microbial diversity. Biochar amended Mediterranean soil, showed reduced leachability and plant availability of HMs (Zn, Cd, Ni, Pb, Cu) (Mendez et al., 2012). Biochar decreased bioavailability and the phytotoxicity of HMs (Cd, Pb, Zn, Cu) through immobilisation of the HMs and improvement of soil nutrients (Park et al., 2011; Rodriguez et al., 2019; Taghlidabad and Sepehr, 2018; Yang et al., 2021a).

### 7.3 Biochar effect on PTEs in wastewater

In a comparative study, comparing use of biochar and precipitation methods on HMs in water, biochar proved effective with 100% HMs removal while precipitation method had 75–80% efficiency (Chughtai and Kashif, 2017). Treatment of wastewater with a biochar-biofilter resulted in an improved physicochemical property of the wastewater and a great reduction in the HMs (Cd, Cr, As, Pb, Zn, Cu) concentration (Das et al., 2021b). Thallium was effectively removed from wastewater using biochar from watermelon rinds (Li et al., 2019a); Pb and Cd ions removed by coffee husk biochar (Quyen et al., 2021); Pb, Fe, Cd, and Cu ions removed by rice husk biochar (Roha et al., 2021; Sanka et al., 2020); sunflower seed husk biochar removed Cu ions (Saleh et al., 2016); coconut shell biochar adsorbed Pb and Cd in wastewater (Wu et al., 2021); and paper mill sludge biochar removed As, Zn, and Cu from wastewater (Xu et al., 2021).

The impact of co-contamination and metal-metal interaction in HMs contaminated wastewater should be considered when using biochar. Co-contamination of HMs in WWTPs can affect their adsorption to biochar as was observed in a study by Caporale et al. (2014). In single contamination of the HMs (Cr, Pb, and Cu), Cr and Pb were effectively adsorbed by the biochar but in a binary contamination, Pb inhibited the adsorption of the other HMs (Caporale et al., 2014). In a ternary contamination, reasonable amount of Cu was adsorbed indicating the effect of competitive sorption, metal interactions, and biochar binding sites. In a similar study, Pb and Cd were adsorbed in a single system but the adsorption of Cd was inhibited in a binary system with no effect on Pb sorption (Ni et al., 2019).

## 8 Challenges and limitations of biochar application

The use of biochar as amendment for soil and composting can cause possible contamination if the biochar contains contaminants (Godlewska et al., 2021; Guo et al., 2020b; Qadeer et al., 2017). Biochar from various plant and animal sources contain PAHs (Fabbri et al., 2013; Quilliam et al., 2013), PCBs (Luo et al., 2014), dioxins (Luo et al., 2014) and PTEs (Gondek et al., 2014; Buss et al., 2015; Qiu et al., 2015) contaminants which depends on the feedstock and production conditions. The contaminant and nutrient content of the biochar feedstock has a significant impact on speciation and distribution of contaminants in biochar (Adanez-Rubio et al., 2021). Freshly produced PAHs-contaminated biochar released PAHs in soils and the level of PAHs released decreased with aging (Sigmund et al., 2017). The pattern of PTEs leaching observed in sewage sludge biochar is similar to what is obtainable with the non-pyrolyzed biosolids (Bialowiec et al., 2020). This implies that conversion of biosolids to biochar has no significant impact on the reduction of PTEs. Sewage sludge-derived biochar leached HOCs and PTEs, and the leaching was higher with biochar produced at 700 °C (Chen et al., 2019). Leachates from biochar amended soils contained PTEs and 16 USEPA PAHs at levels within EU regulation (Bastos et al., 2014). The levels of PTEs and PAHs in the leachates were found to pose ecological risk when exposed to microbes and Daphnia.

Biochar application increased PAHs concentration in soil which in turn increased the concentration in food crops posing a human health risk (Wang et al., 2019d). The PAHs content of biochar amended soils and risk of exposure is high at early stage of amendment and it is affected by initial biochar quantity and soil carbon content (De Resende et al., 2018). This risk diminishes with duration of biochar in soil (due to aging) as the risk equalled that of control soils, in high and low carbon content soils, after three and six years respectively. The sorption property of biochar can facilitate PAHs persistence which can increase the PAHs accessible fraction with time through biochar aging and desorption. The sorption capacity of biochar decreased markedly in soil within 2 years due to aging, and it was estimated to equal unamended soil within 2.5 years (Ren et al., 2018). Exposure of biochar to sunlight and rain can reduce the cumulative PAHs concentration through volatilisation and desorption of mainly the low molecular weight PAHs (Khalid and Klarup, 2015). Therefore, environmental weathering can lower contaminants level in biochar. High biochar application rate in soils should be avoided to reduce potential health risk and ecotoxicity effect of biochar in soil (Godlewska et al., 2021; Quilliam et al., 2013; Shen et al., 2022).

The dose is the poison. Though contaminants can be found in biochar, under proper production and application, their impact may be negligible (Freddo et al., 2012). Biochar addition at ≤1% had no ecotoxic effect but produced ecotoxic effects at 2.5–5% (Han et al., 2021). Using high dose of biochar does not enhance the effectiveness of PTEs removal from aqueous solutions (Boni et al., 2021). The use of <1% biochar in soil amendment is effective and can prevent certain toxic effects of biochar on organisms (Godlewska et al., 2021). A 0.01%–0.2% biochar was more effective than 0.5%–1.0% biochar amendment in both enhanced and non-enhanced microcosms (Omoni et al., 2020). Therefore, high biochar application may not be necessary to achieve desired result. Biochar application below 1%, can produced desired result and circumvent biochar-induced contamination and toxicity.

## 9 Conclusion and future research prospects

Biochar can reduce contaminant bioavailability thereby decreasing contaminants induced co-selection pressure. This does not eliminate the AR potential of the bacteria but reduces the stress induced by the contaminants which can trigger a protective response in the bacteria, resulting in the expression of resistant genes or resistant mechanisms. This will in turn reduce the abundance of ARGs in the bacteria community with time, as there is no trigger to initiate ARGs expression and transfer. Unavailability of HMs and other contaminants in an environment will increase the shock and susceptibility of bacteria to antibiotics as non-existence of a related environmental stressor will lead to a sublime expression of the resistant genes. This will lead to a greater susceptibility before any possible resistant mechanism is established. Biochar can prevent HGT and can eliminate ARGs carried by MGEs as biochar can sorb MGEs and can destroy genetic materials. This will prevent any HGT through transformation and conjugation hence reducing the maintenance and propagation of ARGs. The modification and adjustment of the bacterial diversity and physicochemical parameters by biochar, and the alteration of these parameters, can lead to a shift from abundance of ARGs host bacterial phylum to a less ARGs carrying bacterial community.

The reports in literature, on the effects of biochar on ARGs is promising, but more work needs to be done to fully grasp how effective this could be. It is important to understand the impacts of biochar properties, contaminants properties, bacteria diversity, environmental factors, and level of contamination on ARGs reduction by biochar. Important questions to research on include,

• Can sorbed PTEs become bioavailable with time due to biochar aging and desorption?
• What is the impact of rhizosphere and plant root exudates on ARGs reduction in vegetated and non-vegetated soils?
• Does the efficacy of biochar in ARGs removal depend on biochar particle size and pyrolysis temperature irrespective of feedstock?
• Since biochar can improve microbial proliferation, under what conditions does biochar effectively control ARGs expression and transfer?
• What are the impacts of bacterial diversity on HMR and ARGs distribution as reduction in HMs bioavailability did not affect HMR genes in some studies?
• How effective is biochar on ARGs in mixed contaminants and mixed HMs contamination?

## Declarations

### Author contribution statement

All authors listed have significantly contributed to the development and the writing of this article.

### Funding statement

This research did not receive any specific grant from funding agencies in the public, commercial, or not-for-profit sectors.

### Data availability statement

Data included in article/supp. material/referenced in article.

### Declaration of interest's statement

The authors declare no conflict of interest.

### Additional information

No additional information is available for this paper.
